# Efficacy of non-surgical management and functional outcomes of partial ACL tears. A systematic review of randomised trials

**DOI:** 10.1186/s12891-022-05278-w

**Published:** 2022-04-08

**Authors:** Michael Giummarra, Loretta Vocale, Matthew King

**Affiliations:** 1 Australasian College of Sport and Exercise Physicians, Melbourne, Australia; 2 School of Health and Biomedical Sciences, RMIT University, 124 Latrobe Street, Melbourne, VIC 3000 Australia; 3 La Trobe Sport and Exercise Medicine Research Centre, La Trobe University, Melbourne, Australia

**Keywords:** Anterior crucial ligament, Partial tear, ACL, Non operative, Rehabilitation

## Abstract

**Background:**

The incidence of anterior cruciate ligament (ACL) injuries represents a large burden of knee injuries in both the general and sporting populations, often requiring surgical intervention. Although there is much research on complete ACL tears including outcomes and indications for surgery, little is known about the short- and long-term outcomes of non-operative, physiotherapy led intervention in partial ACL tears. The primary aim of this study was to evaluate studies looking at the effectiveness of physiotherapy led interventions in improving pain and function in young and middle-aged adults with partial ACL tears. Additionally, the secondary aim was to evaluate the completeness of exercise prescription in randomised trials for physiotherapy led interventions in the management in partial ACL tears.

**Methods:**

A comprehensive and systematic search was performed on six databases (*Medline, CINAHL, EMBASE, PEDro, Scopus*, *SPORTDiscus and Cochrane).* The search strategy consisted of two main concepts: (i) partial ACL tears, and (ii) non-operative management. 7,587 papers were identified by the search. After screening of eligible articles by two independent reviewers, 2 randomised studies were included for analysis. The same two reviewers assessed the completeness of reporting using the Toigio and Boutellier mechanobiological exercise descriptions and Template for Intervention Description and Replication (TIDieR) checklist. Group mean standard deviations (SD) for the main outcomes was extracted from both papers for analysis. Prospero Registration Number: CRD42020179892.

**Results:**

The search strategy identified two studies; one looking at Tai Chi and the other Pilates. The analysis indicated that Tai Chi was significant in reducing pain scores and both Tai Chi and Pilates were found to increase Muscle Peak Torque Strength (MPTS) at 180 degrees. Furthermore, Tai Chi showed a significant increase in proprioception.

**Conclusions:**

Physiotherapy led interventions such as Pilates, and Tai Chi may improve pain, proprioception and strength in young and middle-aged adults with partial ACL tears, however full scale, high-quality randomised studies are required with long term outcomes recorded.

**Supplementary Information:**

The online version contains supplementary material available at 10.1186/s12891-022-05278-w.

## Introduction

Injuries of the anterior cruciate ligament (ACL) have been estimated in the general population to be 68.6 per 100,000 person-years [1]. This represents a large burden in both professional and amateur athletes, accounting for approximately 20% [2] to 50% [3] of all knee injuries. Of these, partial ACL tears represent 10% to 28% of all isolated ACL injuries [4]. In Australia alone, there has been an increasing trend over the recent years to proceed to ACL reconstructions with operations increasing by 43% within the last decade [5].

Anatomically, the ACL consists of two bundles which are named according to their tibial attachment. The antero-medial (AM) bundle, taut in flexion, is primarily responsible for restraining anterior tibial translation (anterior drawer test). The postero-lateral (PL) bundle on the other-hand, is taut in extension and is primarily responsible for rotational stability (pivot shift test) [6].

Diagnosis of partial ACL tears is often made by a combination of clinical assessment and magnetic resonance imaging (MRI) findings [7–10] with a general focus on the presence of a soft or delayed end-point on Lachman’s testing and high MRI signal within the ACL structure. At times, clinical diagnosis of ACL tears may prove difficult as patients may display conflicting clinical signs such as a positive Lachman’s test but a negative pivot shift test. Partial ACL tears are a heterogenous group, and consist of 3 main injury patterns: isolated AM bundle, isolated PL bundle or a blended partial injury of both bundles [11]. Clinical diagnosis of partial ACL tears remains difficult. Not only are the clinical tests limited to operator ability and experience, but their validity is also unknown, with specificity and sensitivity only evaluated in complete ruptures. When assessing for complete ruptures, the sensitivity and specificity of the Lachman’s test are reported as being 0.86 and 0.91, respectively. The pivot shift on the other hand has been shown to have a high specificity (0.97–0.99), although it is not overly sensitive; (0.18–0.48) as the Lachman’s test [12]. A recent systematic review of partial anterior cruciate ligament tears reinforced the absence of a positive pivot shift test in these types of injuries [13]. Furthermore, arthroscopic examination revealed these partial tears to have between 25-75% of continuous fibres across the studies [13]. Although not routinely employed due to ethical limitation, diagnosis via arthroscopy remains the gold standard for the diagnosis of both partial tears and complete ruptures [4].

There is no current consensus on ‘standard care’ in the treatment of partial ACL tears, and treatment is often tailored to the individual depending on age, sex, level of sport/activity and other concomitant knee injuries at the time i.e. meniscal tear. Most often, the treatment of partial ACL tears involves surgery [5]; with surgical management of such tears includes bundle sparing, augmentation, or complete reconstructions [11]. Pujol’s review showed that patients had good short and medium term functional results when they limit their sporting activities, with 52% of patients retuning to the pre-injury levels of sporting activity after a partial ACL tear [13].

Thus the primary aim of this systematic review was to assess the current literature into the outcomes of non-operative management of partial ACL tears. Furthermore, in order to assist in the clinical interpretability of the outcomes investigated, we also aimed to evaluate the reporting quality of the investigated interventions to determine their reproducibility in the clinical setting.

## Materials and methods

The protocol for this systematic review was registered on the PROSPERO register (http://www.crd.york.ac.uk/PROSPERO/; registration number CRD42020179892) and followed the Preferred Reporting Items for Systematic Reviews and Meta-Analyses statement [14].

### Search strategy

A systematic search was conducted in the following databases from inception to February 2022: *Medline, CINAHL, EMBASE, PEDro, Scopus, SPORTDiscus,* and *The Cochrane Library (including CENTRAL)*. The search strategy consisted of two main concepts; (i) partial ACL tears, and (ii) non-operative management, with MESH terms and keywords adapted to individual databases as required. Primary key words included ‘partial ACL’, ‘anteromedial’, ‘posterolateral’, for the population and ‘non-operative’, ‘conservative’, ‘physiotherapy’ and ‘rehabilitation’ for the intervention (Supplementary A). Articles were imported into Endnote Reference Management Software (Version X9) for eligibility screening and removal of duplicates.

### Inclusion and exclusion criteria

Eligible studies included randomised trials involving humans of any age with a partial ACL tear (either isolated AM or PL bundles) diagnosed via MRI, who were managed with non-operative interventions. Included studies must have collected at least one of the following; (i) functional (i.e. hop test battery) outcomes; (ii) impairment measures (such as quadriceps strength); or (iii) patient-reported outcome measures (IKDC 2000, [15]) and compared them to an independent control group. Controls were deemed individuals who did not have rehabilitation or surgery and/or a wait and see group. Studies evaluating surgical intervention or those that had diagnosed patients via arthroscopic investigation were excluded. Reviews, case studies, conference abstracts and protocol papers, as well as non-English language publications, were excluded.

### Screening

Titles and abstracts of potentially eligible studies in the Endnote library were independently screened by two reviewers (MVG and MGK), with disagreements resolved by consensus, or a third reviewer (LV) independently reviewing the paper. After title and abstract screening, full-text articles of the retained studies were reviewed to determine their eligibility. Reference lists of included articles were screened and forward citation tracking completed to identify additional eligible articles that had not been identified in the initial search.

### Risk of Bias (RoB) assessment – Physiotherapy Evidence Database (PEDro)

An independent assessment of RoB was not performed by the reviewers as all articles included in this review contained PEDro scores; which is an instrument used to assess the RoB of trials of physiotherapy interventions [16]. The PEDro scale is based on the Delphi list [17] and encompasses psychometric properties allowing for quality assessment of randomised clinical trials for conducting systematic reviews based on expert consensus. PEDro scores for clinical trials range from 0 to 10 with higher scores indicating less RoB.

### Patient and Reported Outcomes Measures (PROM’s) and Assessor Objective Measures (AOM’s)

Both included studies used a patient-reported outcome measure (PROM) and assessor objective measures (AOM) as the primary outcome measure. The PROMs used included the Visual Analogue Scale (VAS), the Lysholm Knee Scale (LKS), the International Knee Documentation Committee 2000 Questionnaire (IKDC) and the Cincinnati Knee Scale (CKS).

The VAS was first described by Freyd [18] in 1923, consisting of a straight line with endpoints labelled “no pain” at one end and “unbearable pain” at the other end. Patients are then asked to mark their pain on the straight line between the two endpoints. The distance between ‘no pain at all’ and the patients mark indicates the level of the patients’ pain. The VAS does have good test–retest reliability [19].

The LKS is used to examine a patients knee-specific symptoms. This scale is scored between 0 to 100, with 25 points attributed each to pain and instability, 15 points to locking, 10 points each to swelling and stair climbing, and 5 points each to limping and use of a support and squatting. The LKS demonstrates acceptable test–retest reliability, internal consistency, and construct validity [20].

The IKDC is and 18-item questionnaire designed to document knee ligament injuries in three domains of (i) symptoms, (ii) sports, and daily activities, and (iii) current knee function and knee function prior to knee injury (not included in the total score) [21]. The IKDC demonstrates adequate reliability and validity [15].

The CKS is scored between 120 to 420 and is a functional assessment used in sport, based on six abilities including walking using stairs, squatting and kneeling, straight running, jumping and landing, and twists cuts and pivots. The CKS demonstrates high viability, reliability and responsiveness [22, 23].

In all of the above PROMs with the exception of the VAS, a higher score indicates a better outcome.

The AOMs used in the studies were Muscle Peak Torque Strength (MPTS) at both 60 degrees of flexion and extension as well as both 180 degrees of flexion and extension. Proprioception was also measured in one study. Both MPST and proprioception was assessed using the Biodex System 4-Pro (Biodex Inc., Shirley, NY, USA). The MPST results were recorded as the isokinetic peak torques for knee flexion and extension muscle strength; with a higher recording being more favourable. Proprioception on the other hand recorded the average error of the patients’ scores, so a lower score indicates a more favourable result.

### Data extraction

#### Primary aim

Information on study design, sample characteristics (e.g., age, sex, inclusion criteria), and outcome measures were extracted and entered into an Excel spreadsheet by one reviewer (MVG) with a random selection of 50% of the extracted data checked by another reviewer (MGK). Group mean and standard deviation (SD) of the main outcomes were extracted for data analysis. Where data was presented in graphical form only, images were digitised and data extracted. If necessary, authors were contacted for further information to confirm eligibility and facilitate accurate data extraction.

#### Secondary aim

Data were independently extracted by two reviewers (MVG and MGK) for each item of the TIDieR checklist and Toigo & Boutellier’s [24] exercise descriptors. If reference was made to additional details in an appendix or supplement, then the relevant information was extracted from these additional sources. A checklist of both complete and incomplete items was compiled from the extracted information of each study. Items were considered complete and scored one point if they were clearly and unambiguously described, to an extent which would allow them to be replicated. Scores were compiled for each study, for the number of complete items on each of the checklists (Toigo & Boutellier’s [24]and TIDieR). Data extraction was completed in a specifically developed Excel spreadsheet. Disagreements were addressed through a consensus discussion.

### Data analysis

#### Primary aim

Standardized mean differences and 95% confidence intervals of between-group changes in post-rehabilitation outcomes were calculated to address the primary aim. This was conducted by dividing the difference between the groups by the pooled SD. Due to the heterogenous nature of the outcomes, studies were not pooled in a meta-analysis. Instead, a qualitative synthesis was conducted using Cohens criteria, and interpreted on a per study basis, with an standardized mean difference (SMD) ≥ 0.8 defined as a large effect, > 0.5 and < 0.8 defined as moderate and ≤ 0.5 as small [25].

#### Secondary aim

To address the secondary aim of the review, included studies’ exercise interventions were assessed against both the TIDieR checklist and the Togio and Boutellier [24] mechanobiological exercise descriptors. The TIDieR checklist was designed with the aim of providing consistency in research translation. Furthermore, the omission of vital information in the description of research method was illustrated by the EQUATOR network and is a major issue in current health research publications [26]. Togio and Boutellier [24] outlines 13 important mechanobiological descriptions, which provide a framework to standardise the design description of resistance exercise interventions.

## Results

### Search strategy and study selection

**Fig. 1:**
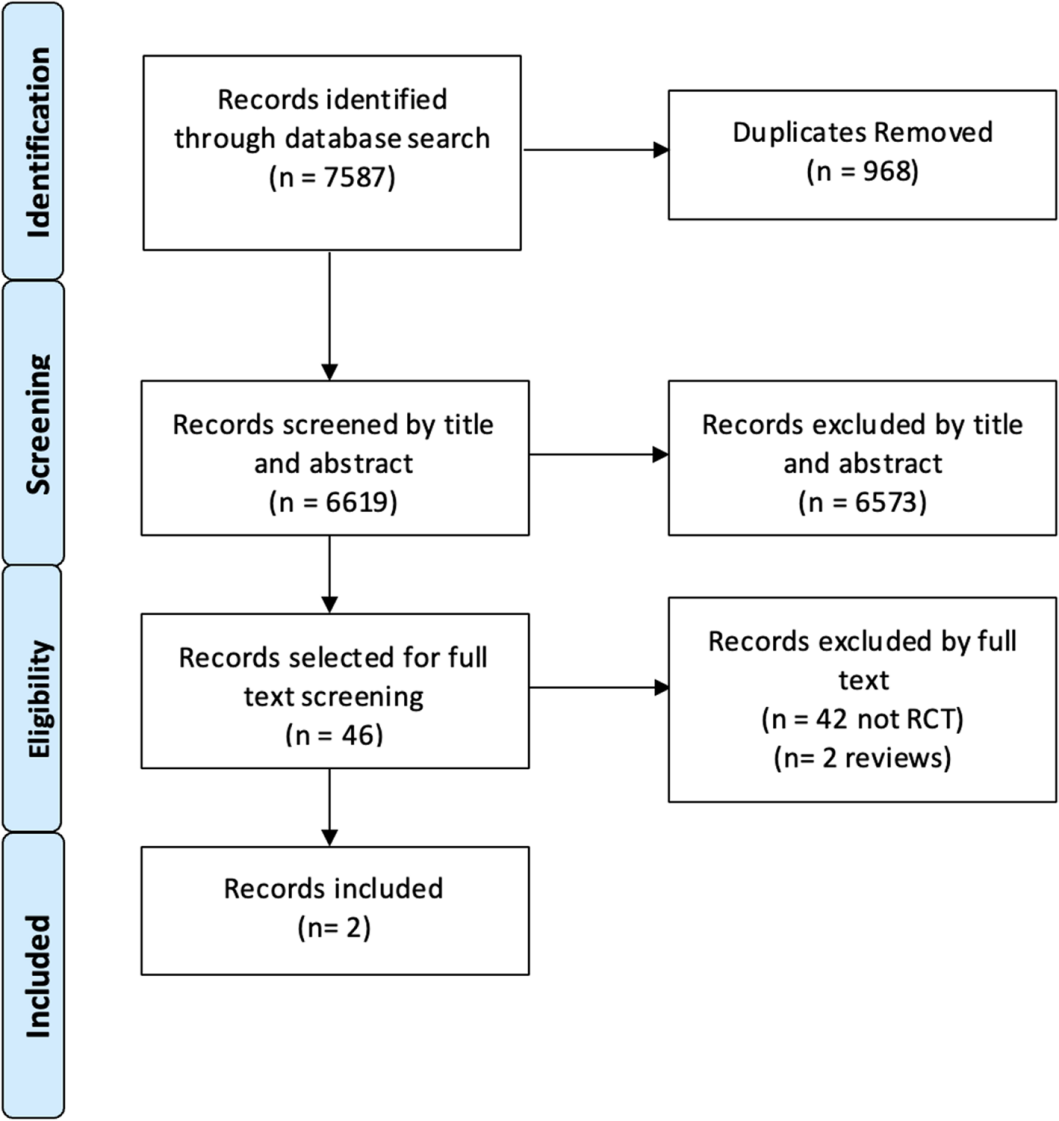
PRISMA flow diagram depicting how articles were selected for review

The search strategy identified 7,587 articles for evaluation (Fig. 1). Following the removal of duplicates, 6,619 articles were evaluated for inclusion. Title and abstract screening excluded 6,573 studies. The remaining 46 full-text articles were assessed for eligibility with two studies meeting the inclusion/exclusion criteria. Both randomised studies investigated the functional outcomes of partial ACL tears when treated non-operatively with a mode of physiotherapy—characteristics of the included studies presented in Table 1.

**Table 1:** Characteristics of included studies

				**Participants (Control Group)**				**Participants (Intervention Group)**				**Intervention Details**
**Reference**	**RCT**	**Inclusion/Exclusion criteria**	**Diagnostic Criteria**	***N***	**Age (years): M (± SD), range**	**BMI (kg/m2)**	**Injury Characteristics**	***N***	**Age (years): M (± SD), range**	**BMI (kg/m2)**	**Injury Characteristics**	**Intervention**
Buyukturan et al. [27]	single-blinded RCT	*Inclusion:* sedentary occupation or low activity level and had VAS scores < 3. Exclusion: participants with meniscus injuries, meniscus tears, chondral lesions, other ligament laxities, a grade IV injury based on the Lachman test, generalised laxity, and/or a BMI > 30 kg/cm2 were excluded from the study	Conducted by physician based on the patient histories, physical examinations, and diagnostic imaging	29 (15 females, 14 males)	24.7 (± 6.2) years	24.9 + -5.7	Dominant side R/L, 26/3; Involved dominant/Non-dominant, 22/7; Duration of the symptoms, 21.8 ± 4.8 months; Education status 13.7 ± 1.7 years	29 (13 females, 16 males)	26.1 (± 7.4) years	24.6 + -4.1	Dominant side R/L, 25/4; Involved dominant/Non-dominant, 20/9; Duration of the symptoms, 20.1 ± 8.6 months; Education status 14.2 ± 2.6 years	3 × 60 min sessions for 24 weeks of Tai Chi -Yang style (10 forms) by experienced instructor (> 5 years’ experience)
Celik et al., [28]	single-blinded RCT	*Inclusion:* sedentary occupation or low activity level, required conservative care rather than surgery, and had VAS scores < 3. Exclusion: participants with meniscus injuries, meniscus tears, chondral lesions, other ligament laxities, a grade IV injury based on the Lachman test, a generalised laxity, and/or a BMI > 30 kg/cm2 were excluded from the study	Conducted by physician based on the patient histories, physical examinations, and diagnostic imaging	26 (24 females, 2 males)	25.9 (± 7.5) years,^a^ 22–45 years	25.8 ± 4.2	Dominant side R/L, 20/6; Involved dominant/Non-dominant, 14/10; Duration of the symptoms, 6.2 ± 1.2 months	24 (22 females, 2 males)	25.2 (± 5.3) years, 20–43 years	25.5 ± 5.1	Dominant side R/L, 20/4; Involved dominant/Non-dominant, 16/8; Duration of the symptoms, 5.4 ± 2.4 months	3 × 60-min Pilates classes per week for 6 weeks run by a trained physical therapist. Home programme was given to participants to engage in after the initial 6 weeks until the end of the study

^a^Ages reported in table, note this is different to the ages reported in the body of the text of the article. Values expressed as mean ± SD, *M* mean, *SD* standard deviation

### Risk of Bias within studies

RoB assessments of the randomised clinical trials were conducted to ascertain if the studies satisfied the final inclusion criteria and determine the study quality in.

relation to the objective of the current review. Both included studies were already attributed to a PEDro score. Buyukturan et al. [27] focused on Tai Chi intervention with a PEDro score of 6/10, whereas Celik and Turkel [28] incorporated a Pilates regime scored 4/10 on the PEDro scale. Both studies were designed with random allocation, and baseline comparability and both went on to provide between-group comparisons with point estimates and variability. Buyukutran et al. [27] employed blinded assessors to perform the objective measurements with adequate follow up. In both studies, there was no concealed allocation, nor blinding of subjects or therapists. Furthermore, there was no intention to treat analysis performed in either of the studies (see Table 2 below).

**Table 2:** Risk of Bias assessment (PEDro scrore)

Author	Eligibility Criteria	Random Allocation	Concealed Allocation	Baseline Comparability	Blind Subjects	Blind Therapists	Blind Assessors	Adequate Follow-up	Between Group Comparisons	Point estimates and variability	Total Score
Buyukturan	Yes	Yes	No	Yes	No	No	Yes	Yes	Yes	Yes	6/10
Celik	Yes	Yes	No	Yes	No	No	No	No	Yes	Yes	4/10

Eligibility criteria item does not contribute to total score] *This score has been confirmed*

### Study characteristics

A total of 123 participants were randomised into control and intervention groups. Across both studies, there was a total of 60 participants allocated to the control group and 63 participants allocated to the intervention group. Both studies were of RCT design. A total of 108 participants (35 male, 73 female) remained at follow-up after dropouts. Mean participant age in the intervention group was 25.6 ± 8.3 years compared to 25.3 ± 6.9 years in the control group. The mean body mass index (BMI) of the intervention group was 25.05 and 24.8 in the control group. The dominant right side was affected in 85% and 84% of intervention and control subjects, respectively. In total, the right side was affected in 68% of all intervention subjects compared to 66% of all control subjects. The average symptom duration was 22 weeks in intervention groups and 23 weeks in the control groups.

Methods for diagnostic inclusion criteria were identical in both studies, including clinical diagnosis by a specialist physician, history, physical examination, diagnostic imaging, aged 25–45 years, sedentary lifestyle with a low level of activity and Visual Analogue scale (VAS) < 3. Celik and Turkel [28] specified an additional inclusion criterion being that of requiring conservative treatment rather than surgery.

Study characteristics are presented in Table 1 and include any additional information provided by study authors.

### Outcomes of non-operative management of partial ACL tears

Between-group SMDs were generated for physiotherapy led intervention groups and compared with the comparator control group who did not undergo rehabilitation. All baseline scores in outcomes investigated were non-significant between the intervention group and controls, with the exception of MPTS at 60 degrees extension in the Tai Chi study [27]. The active intervention group were significantly weaker at baseline compared to the control group (Table 3).

**Table 3:** Between group standard mean differences (SMDs)

							MPTS			
		IKDC 2000	LKS	Pain (VAS)	Proprioception	CKS	Extension 60 degrees	Flexion 60 degrees	Extension 180 degrees	Flexion 180 degrees
Buyukturan et al. [27]	Baseline	-0.05 [-0.56, 0.47]	-0.30 [-0.82, 0.22]	-0.15 [-0.67, 0.36]	0.39 [-0.13, 0.91]		-0.67 [-1.20, -0.14]	0.34 [-0.18, 0.86]	-0.18 [-0.69, 0.34]	0.38 [-0.14, 0.90]
	Follow up	0.25 [-0.26, 0.77]	-0.10 [-0.61 0.42]	-2.17 [-2.83, -1.52]	-1.33 [-1.90, -0.75]		0.56 [0.04, 1.09]	0.51 [-0.02, 1.03]	1.32 [0.75, 1.89]	0.55 [0.03, 1.08]
Celik et al. [28]	Baseline		0.36 [-0.92, 0.20]			-0.10 [-0.65, 0.46]			-0.19 [-0.74, 0.37]	-0.03 [-0.59, 0.52]
	Follow up		-0.29 [-0.84, 0.27]			0.37 [-0.19, 0.93]			0.63 [0.06, 1.20]	0.35 [-0.21, 0.90]

Reported as Standard Mean Difference [95% confidence interval]

*IKDC* International Knee Documentation Committee Questionnaire, *LKS* Lysholm Knee Scale, *VAS* Visual Analogue Scale, *CKS* Cincinnati Knee Scale, *MPTS* Muscle Peak Torque Strength

### Between-group comparisons of PROMs for physiotherapy led interventions compared with control

**Fig. 2:**
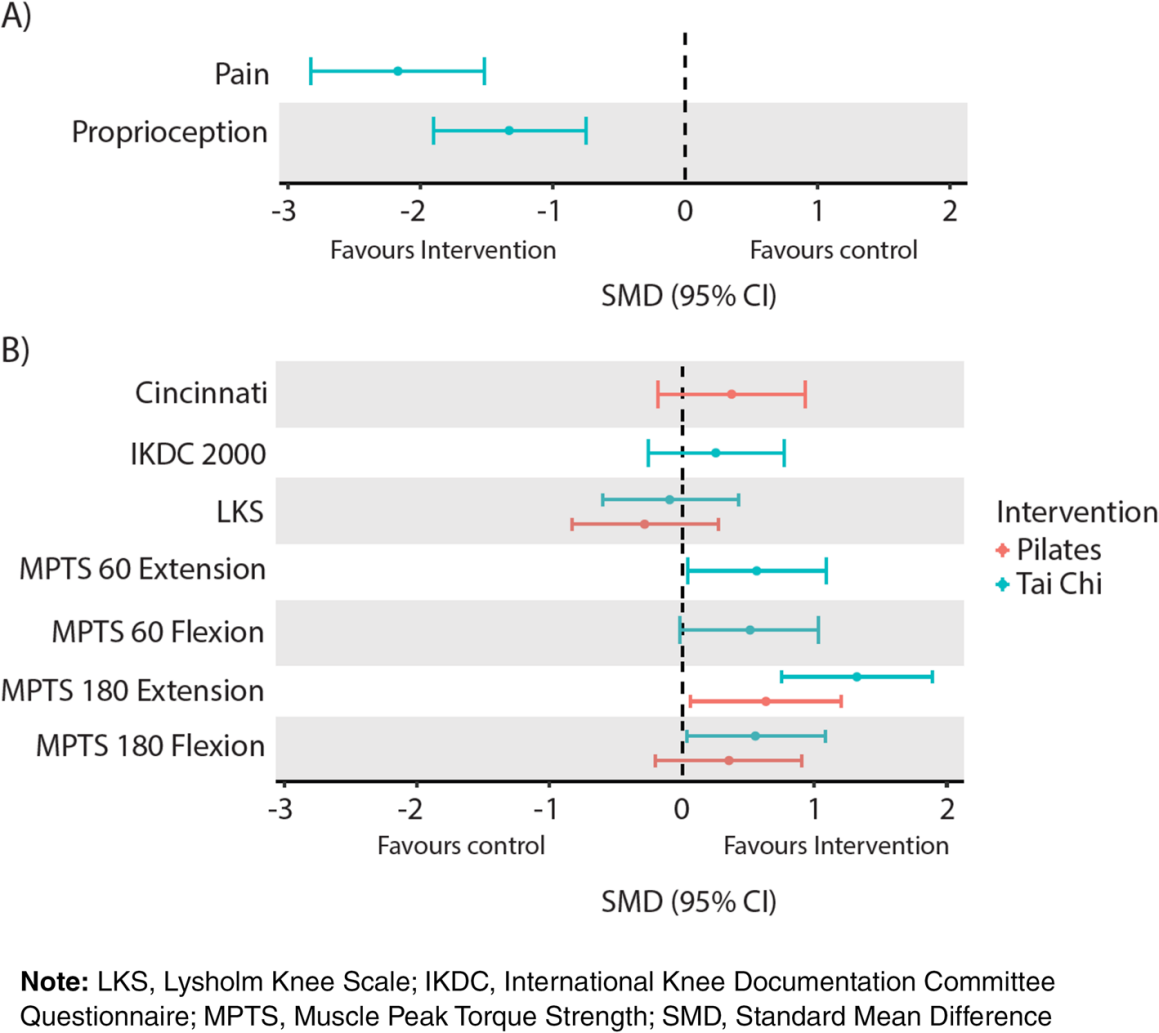
SMD results for between-group comparisons at follow up. This figure shows (**A**) outcomes where a negative score in favourable in the intervention group and (**B**) outcomes where a positive score in favourable in the intervention group

Tai Chi was found to significantly reduce pain scores at follow up compared to controls (large effect, SMD -2.17, 95%CI -2.83 to -1.52) (Table 3, Fig. 2). No additional PROMs (IKDC, LKS and CKS) were significantly different across the two groups at follow up (Table 3, Fig. 2).

### Between-group comparisons of AOMs for physiotherapy led interventions compared with control

Both Tai Chi and Pilates were found to significantly increase MPTS at 180 degrees of extension at follow up compared to controls (Tai Chi: large effect, 1.32, 0.75 to 1.89; Pilates: moderate effect, 0.63, 0.06 to 1.20) (Table 3, Fig. 2). Furthermore, Tai Chi showed a significant increase in proprioception (large effect -1.33, -1.90 to -0.75), MPTS at 60 degrees of extension (moderate effect, 0.56, 0.04 to 1.09) and 180 degrees of flexion (moderate effect 0.55, 0.03 to 1.08) (Table 3, Fig. 2) at follow up compared to controls. No significant difference were observed in MPTS at 60 degrees of flexion and MPTS at 180 degrees of flexion in the pilates group compared to controls at follow up (Table 3, Fig. 2).

### Reporting quality and reproducibility of Tai Chi and Pilates in the clinical setting

**Fig. 3:**
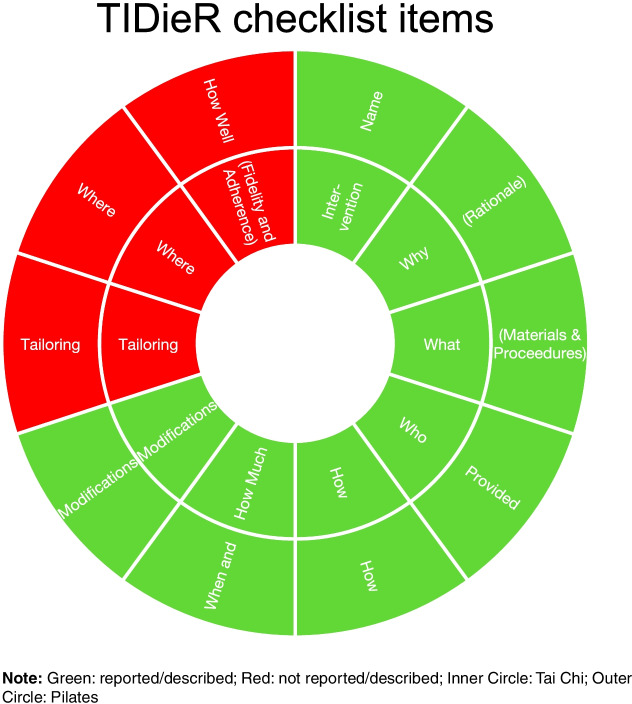
TIDieR checklist items for each study. Template for intervention description and replication

**Fig. 4:**
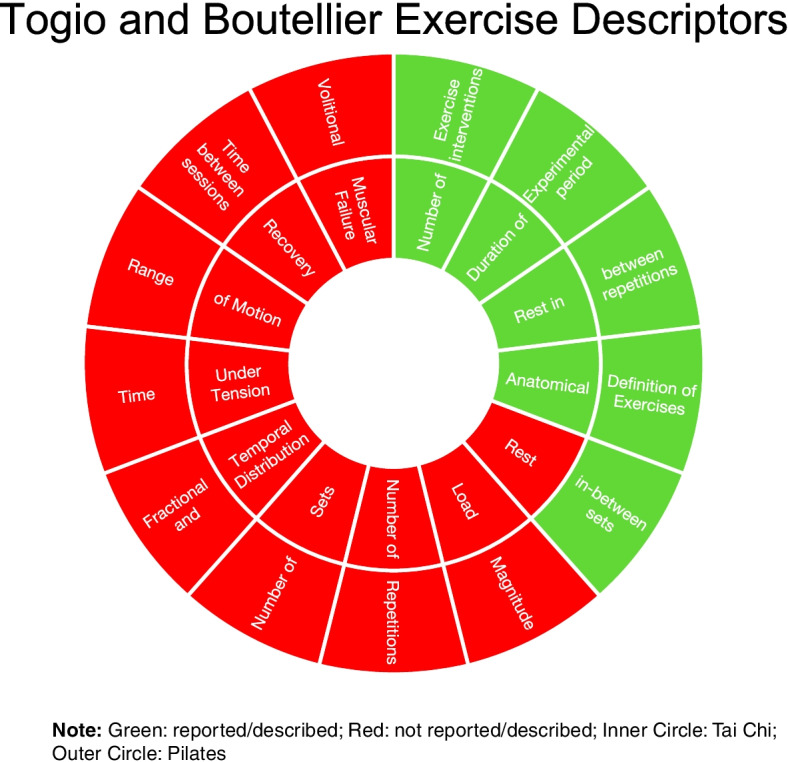
Reporting for each of the Togio and Boutellier exercise descriptors

Both studies were assessed against the TIDieR checklist and Toigo and Boutellier exercise descriptors [24] (Figs. 3 and 4). Neither study provided complete information for all of the items, with both failing to provide the location where the intervention occurred and how well the study was performed (adherence and fidelity). Furthermore, Celik and Turkel [28] failed to provide any tailoring to the subjects whereas Buyukturan et al. [27] did.

The most-reported items from the Toigo and Boutellier [24] exercise descriptors common to both studies were “number of exercise interventions”, “duration of the experimental period” the “rest in between repetitions” and “anatomical definition of the exercise”. Celik and Turkel [28] provided “rest in between sets” whereas Buyukutran et al. [27] did not. Neither study described “load magnitude”, “number of sets”, “fractional and temporal distribution”, “time under tension”, “volitional muscle failure”, “range of motion”, “recovery time in between sessions”.

## Discussion

This systematic review aimed to identify studies in the literature which evaluate the effectiveness of physiotherapy led interventions to improve both pain and function in young and middle-aged adults with partial ACL tears, compared to wait and see groups. Furthermore, this review evaluated the reproducibility of the included studies by placing them through a stringent study design criteria used to evaluate exercise intervention and randomised studies.

The two interventions studied demonstrated efficacious outcomes of impairment based measures (pain, proprioception, MPTS) but not on patient-reported quality of life and functional restrictions (LKS, IKDC 2000, CKS). Both Tai Chi and Pilates showed significant improvements in the quadriceps extensor strength in the intervention groups at follow up. This is important as strong quadriceps are an essential muscle group for ACL rehabilitation and in the prevention of further ACL injury [29]. Furthermore, Tai Chi showed a significant increase in proprioception in the intervention group at follow-up. This is favourable in ACL rehabilitation as previous studies have shown that proprioceptive feedback is important in both functional outcomes and ACL stability [30]. Pain was the only patient-reported outcome to show significant improvement at follow up in the Tai Chi intervention group. Unfortunately, this was not measured in the Pilates group.

Both interventions did not demonstrate a significant improvement in patient-reported quality of life or functional outcomes when compared to the control groups. Such a finding is consistent with previously published guidelines indicating that an increase in impairment-based measures does not necessarily translate to improvements in activity and participation restrictions [31]. Incorporation of specific retraining of known activity restrictions may have improved these outcomes further and should be investigated in future research.

The quality of the included studies was fair, with both studies sitting within the accepted standard deviation range of the average Pedro score (5.1 ± 1.5) [32]. Both studies adequately nominated eligibility criteria and performed random allocation with baseline comparability. However, neither provided concealed allocation nor blinding of the subjects or therapists. Such detection bias may have been difficult to control in these studies as the control group being a wait and see approach did not include alternative intervention prevented the aversion of such bias.

Reporting of exercise intervention programs in both studies evaluating the efficacy of exercise therapy for partial ACL tears was sufficient, with enough detail to allow full replication by other researchers in the future. This reporting was highlighted by both the commonly accepted TIDieR checklist and Toigo and Boutellier [24] criteria, the latter which relate specifically to resistance training interventions. Both studies satisfied the majority of the TIDieR checklist items, with the exception of location and how well the study was performed (adherence and fidelity). These items would not necessarily prevent future replication of the study, however, may be ideal for future researches to include in their study design for completeness. Furthermore, the Pilates study failed to provide any tailoring to the intervention group, which may have affected the reporting outcomes. Not reporting these items may affect the accurate interpretation of outcomes and could prevent future interventions from tailoring approaches to increase fidelity and adherence.

We note that the Toigo and Boutellier [24] exercise descriptors are more tailored to strength—type exercises, of which both Pilates and Tai Chi are not. Therefore, providing a complete list of descriptors was not possible by either study. Interestingly, we note that muscle strength, in particulate knee extensions strength, did improve in both studies. It is possible that although not prescribed to standard strength training principals [33], the presence of resistance type movements in the rehabilitation protocols could provide enough load to result in an increase in strength when compared to wait and see. Future research should establish the size of effects of differing strength and resistance rehabilitation protocols to adequately inform clinicians on what exercises and/or movements will provide the greatest efficacious outcome for their intended aim.

There are limitations associated with the review that require acknowledgement. Firstly, this review only identified two randomised studies looking at non-operative intervention in the management of partial ACL tears. This highlights the lack of research in non-operative interventions for partial ACL tears prompting a need for further investigations in this area. Furthermore, there was only one study each looking at Tai Chi and Pilates. Both these interventions, although non-operative, have some heterogeneity in their exercise and rehabilitation protocols and hence may not be translatable across the two studies. Drawing absolute conclusions is therefore difficult and limited to outcomes and populations studied. Secondly, RoB was moderate in both studies suggesting that the study designs could have been improved; this, along with the paucity of long-term data makes drawing absolute conclusions difficult and should be considered when interpreting the results. Finally, the Togio and Boutellier [24] descriptors are specific to resistance training and therefore may not be directly applicable to the intervention therapies within this review, which composed of mainly stretching, flexibility and endurance.

## Conclusions

Overall, the main findings from this review suggest that non-operative, exercise intervention, appears to demonstrate efficacious outcomes in patients with partial ACL injuries, when compared to a wait and see control. However, the review highlights the paucity of information available on non-surgical management of partial ACL tears. Future research should evaluate partial ACL outcomes with more stringent designs, and longer follow up time periods.

## Supplementary Information

**Additional file 1.**

## Data Availability

Not applicable.

## Abbreviations

ACL: Anterior cruciate ligament
AM: Antero-medial
AOM: Assessor objective measures
BMI: Body mass index
CINAHL: Cumulative Index to Nursing and Allied Health Literature
CKS: Cincinnati Knee Scale
IKDC: International Knee Documentation Committee
Kg/cm^2^: Kilogram per Square Centimetre
LKS: Lysholm Knee Scale
M: Mean
MPTS: Muscle Peak Torque Strength
MRI: Magnetic resonance imaging
*N*: Population size
PEDro: Physiotherapy Evidence Database
PL: Postero-lateral
PROM: Patient and Reported Outcomes Measures
RCT: Randomized control trial
RoB: Risk of bias
SD: Standard deviation
SMD: Standard mean difference
TIDieR: Template for Intervention Description and Replication
VAS: Visual Analogue Scale
