# Supplementary material for: Efficacy of non-surgical management and functional outcomes of partial ACL tears. A systematic review of randomised trials
Source: BMC Musculoskelet Disord. 2022 Apr 8;23:332. doi: 10.1186/s12891-022-05278-w (PMC8991495; doi:10.1186/s12891-022-05278-w)
Supplement: Supplementary file 1 — Additional file 1. [file 12891_2022_5278_MOESM1_ESM.docx]

**Supplementary A: MESH terms and keywords used for Medline Search**

| 1. exp Anterior Cruciate Ligament Injuries |
| --- |
| 1. "partial anterior cruciate lig*".mp. |
| 1. "partial ACL".mp |
| 1. "anteromedial ACL".mp. |
| 1. "anteromedial anterior cruciate lig*".mp. |
| 1. "posterolateral ACL".mp |
| 1. "posterolateral anterior cruciate lig*".mp. |
| 1. 1 or 2 or 3 or 4 or 5 or 6 or 7 |
| 1. exp Physical Therapy Modalities/ |
| 1. exp Rehabilitation/ |
| 1. exp Conservative Treatment/ |
| 1. exp Watchful Waiting/ |
| 1. "rehab*".mp. |
| 1. "physiothe*".mp. |
| 1. conservative.mp. |
| 1. "non op*".mp. |
| 1. "non surg*".mp. |
| 1. 9 or 10 or 11 or 12 or 13 or 14 or 15 or 16 or 17 |
| 1. 8 and 18 |
